# Predictive Value of a Noninvasive Serological Hepatic Fibrosis Scoring System in Cirrhosis Combined with Oesophageal Varices

**DOI:** 10.1155/2018/7671508

**Published:** 2018-08-14

**Authors:** Feiyue Zhang, Tong Liu, Pan Gao, Sujuan Fei

**Affiliations:** ^1^Department of Gastroenterology, Xuzhou Medical University, Xuzhou, Jiangsu 221000, China; ^2^Department of Gastroenterology, The Affiliated Hospital of Xuzhou Medical University, Xuzhou, Jiangsu 221000, China

## Abstract

**Objective:**

In recent years, the noninvasive serological scoring system has become a research hotspot in predicting hepatic fibrosis and has achieved good results. However, it has rarely been applied to the prediction of oesophageal varices. The aim of the study was to evaluate the predictive value of the four following scoring systems in cirrhosis combined with oesophageal varices: aspartate and platelet ratio index (APRI), aspartate aminotransferase-alanine aminotransferase ratio (AAR), FIB-4, and S index.

**Methods:**

A total of 153 patients with cirrhosis were categorized into groups with or without oesophageal varices. In addition, cirrhosis patients with oesophageal varices were further divided into mild, moderate, and severe grades. The rank sum test was used to compare the significant differences of APRI, AAR, FIB-4, and S index between the two groups of cirrhosis patients with or without oesophageal varices. A ROC curve was generated to compare the area under the curve of the three groups and to obtain the corresponding optimal prediction value. Moreover, multivariate logistic regression analysis was employed to assess the predictive factors for cirrhosis combined with oesophageal varices.

**Results:**

44 patients had no oesophageal varices and 108 patients had oesophageal varices. Of the 108 patients with oesophageal varices, 43 were mild, 32 were moderate, and 33 were severe. The rank sum test indicated that the APRI, FIB-4, and S index were statistically significant between two groups (*P* < 0.05), while no significant difference was detected in terms of AAR between the two groups (*P* > 0.05). In addition, all four scoring systems were statistically significant between nonoesophageal varices group and severe oesophageal varices group (*P* < 0.05). In the ROC curve of oesophageal varices, the AUC values of APRI, FIB-4, and S index for predicting oesophageal varices were 0.681, 0642, and 0.673, respectively. However, in the ROC curve of severe oesophageal varices, the AUC values of APRI, AAR, FIB-4, and S index were 0.729, 0.648, 0.673, and 0.695, respectively. Multivariate logistic regression analysis indicated that APRI and FIB-4 were predictors of disease progression (*P* < 0.05).

**Conclusion:**

AAR harboured a poor predictive value for oesophageal varices, APRI can be used as a reference index for the prediction of severe oesophageal varices, and the S index harboured potential value in predicting the degree of progression of cirrhosis.

## 1. Introduction

Liver cirrhosis, a common chronic liver disease, is caused by long-term or repeated damage to liver tissue due to one or more causes, leading to diffuse degeneration and necrosis of hepatocytes, regenerative nodules, and fibrous tissue hyperplasia. Portal hypertension is observed in the advanced stage of cirrhosis. When the portal vein pressure increases to a certain degree, oesophageal varices can occur, while, in severe cases, oesophageal variceal bleeding will emerge, which is the most common and severe complication of cirrhosis and cirrhotic portal hypertension, as well as the most common cause of death for cirrhosis [1]. In the 1990s, the mortality rate after the onset of oesophageal variceal bleeding was as high as 50% [2]. With the development and advancements of medical technology and equipment, along with a further understanding of the disease, the mortality rate has decreased; however, new statistics indicate that the 6-week mortality rate is still as high as 20% [3]. Digestive endoscopy is considered the gold standard for the screening and diagnosis of oesophageal varices [4]. However, as an invasive examination, there are potential risks, such as bleeding and anaesthesia accidents during operation, and repeated monitoring can introduce more pain, leading to poor compliance, which makes it difficult to conduct clinical observations on patients with oesophageal varices. In recent years, the exploration of noninvasive methods for predicting oesophageal varices has become a research focus, aiming to selectively assess patients with a relatively high risk of oesophageal varices to further effectively monitor the degree of varices, to administer selective interventions, and to reduce the pain and burden of patients to some extent. Recently, the application noninvasive serological examinations in the prediction and diagnosis of the degree of hepatic fibrosis has become a hotspot. Among them, the classic ones, including APRI and FIB-4 scores, have been recommended by the World Health Organization (WHO) guidelines for assessing hepatic fibrosis [5]. In addition, it has also been shown that AAR is 0.8 in healthy individuals, and AAR > 1 indicates the presence of cirrhosis. Chinese scholars have established an S index in the analysis of risk factors for hepatic fibrosis in chronic hepatitis B virus (HBV), which harbours a higher accuracy in the assessment of hepatic fibrosis in HBV [6]. The above four scoring methods are simple and easy to obtain in clinical practice. However, few studies have applied it in the prediction of cirrhosis and oesophageal varices. Hence, in this study, we aimed to explore and compare the predictive value of the APRI, AAR, FIB-4, and S index scoring systems in oesophageal varices.

## 2. Subjects and Methods

### 2.1. Subject

We retrospectively analysed patients with cirrhosis who were admitted to the Affiliated Hospital of Xuzhou Medical University from January 2010 to September 2017. The inclusion criteria were as follows: ① all patients met the diagnostic criteria for cirrhosis in the Guidelines for Chronic Hepatitis B Prevention and Treatment (2015 Update) by the Chinese Medical Association, 2015 Edition) [7] (gastroscopy and serum biochemical examination were performed after admission). The exclusion criteria were as follows: ① a history of hepatic carcinoma; ② prior hepatic operation and splenectomy; ③ prior transjugular intrahepatic portosystemic shunt; ④ prior sclerotherapy or ligation for oesophageal varices; ⑤ the existence of other factors that might affect the platelet count and spleen size; ⑥ patients with severe heart, lung, and kidney disease; ⑦ patients who have used drugs that inhibit or promote bone marrow haematopoiesis or that decrease portal vein pressure and albumin in the last six months; ⑧ the emergence of upper gastrointestinal bleeding during previous and current admission.

### 2.2. Methods

We recorded the age of the patients and the levels of AST, ALT, albumin, and PLT in peripheral blood. In addition, patients were divided into slight, moderate, and severe groups according to the morphology of oesophageal varices (EV) and the severity of haemorrhage under gastroscopy (shown in Table 1).

### 2.3. Calculation

APRI = (AST/upper limit of normal)×100/PLT (10^∧^9/L); AAR = AST/ALT; FIB-4 = (year of age × AST)/(PLT × the square root of ALT); S index: 1000*∗*GGT/ (PLT*∗* albumin^2^).

### 2.4. Statistical Analysis

SPSS22.0 statistical software was used for all data processing and analysis. The chi-square test was used for enumeration data, the median (quartile) was used for nonnormally distributed measurement data, and the rank sum test was employed for comparisons between groups.* P *< 0.05 represented statistical significance. The combination with or without oesophageal varices in cirrhosis was taken as the demarcation point, and the diagnostic criteria of gastroesophageal varices under gastroscopy were considered the gold standard to investigate whether the four scoring systems of APRI, AAR, FIB-4, and S index were statistically significant between cirrhosis without oesophageal varices and cirrhosis with oesophageal varices. Whether cirrhosis was combined with oesophageal varices or with severe oesophageal varices or not was taken as two demarcation points. The ROC curve was generated, followed by the use of the AUC value to evaluate the predictive accuracy of three scores in cirrhosis combined with oesophageal varices or severe oesophageal varices. The optimal demarcation point was detected, which corresponded to the maximum value by adding sensitivity and specificity on the ROC curve. AUC values > 0.7 indicated clinical value, AUC values of 0.8-0.9 suggested relatively good clinical value, and AUC values > 0.9 indicated very good clinical value. Nonconditional multivariate logistic regression analysis was performed on multiple sets of data. A* P* < 0.05 was considered to represent statistical significance.

## 3. Results

### 3.1. General State

A total of 153 patients with cirrhosis who met the requirements were collected (shown in Table 2). Of them, there were 109 patients with HBV cirrhosis, 11 patients with hepatitis C virus (HCV) cirrhosis, 10 patients with alcoholic cirrhosis, 10 patients with autoimmune cirrhosis, and 13 patients with other causes (drug-induced cirrhosis, mixed cirrhosis, and cryptogenic cirrhosis). There were 44 cirrhosis patients without oesophageal varices (nonoesophageal varices group), 43 with mild oesophageal varices (mild group), 31 with moderate oesophageal varices (moderate group), and 32 patients with severe oesophageal varices (severe group). There was no significant difference in age or gender between patients in each group (all* P* > 0.05).

### 3.2. APRI, AAR, FIB-4, and S Index

Due to the nonnormal distribution of the data, median (M) and quartile range (Q) were used. The group and data of each group are shown in Table 2.

### 3.3. Comparison of the Nonoesophageal Varices Group and Oesophageal Varices Group

APRI: 1.0 (0.6-3.5) versus 2.2 (1.5-3.8); AAR: 1.1 (0.8-1.2) versus 1.1 (0.9-1.4); FIB-4: 102.6 (55.5-562.7) versus 247.8 (130.2-529.4); S index: 0.3 (0.1-1.0) versus 0.8 (0.4-1.8). The rank sum test was used to compare the nonoesophageal varices group and oesophageal varices group, which revealed that APRI scoring system statistically significant (*P* < 0.001), and the *P* values of AAR, FIB-4, and S index were 0.078, 0.006, and 0.001; in other words, all three scoring systems were statistically significant (all* P* < 0.05).

### 3.4. Comparison of the Nonoesophageal Varices Group and Severe Oesophageal Varices Group

APRI: 1.0 (0.6-3.5) versus 2.7 (1.9- 4.2); AAR: 1.1 (0.8-1.2) versus 1.2 (0.9-1.4); FIB-4: 102.6 (55.5-562.7) versus 244.0 (153.3-518.1); S index: 0.3 (0.1-1.0) versus 0.8 (0.4,1.9). The rank sum test was used to compare the nonoesophageal varices group and severe oesophageal varices group, which revealed *P* values of 0.001, 0.026, 0.009, and 0.003, respectively; in other words, all four scoring systems were statistically significant (all* P* < 0.05).

### 3.5. Predictive Value of APRI, FIB-4, and S Index in Oesophageal Varices (Shown in Figure 1)

ROC curve analysis showed that the AUC values of APRI, FIB-4, and S index in predicting oesophageal varices were 0.681, 0.642, and 0.673, respectively (shown in Table 3). The optimal demarcation point of blood APRI in predicting cirrhosis combined with oesophageal varices was 1.29, and the corresponding sensitivity and specificity were 0.815 and 0.600, respectively. The optimal demarcation point of FIB-4 in predicting cirrhosis combined with oesophageal varices was 103.7, with a corresponding sensitivity and specificity of 0.843 and 0.511, respectively. The optimal demarcation point of the S index in predicting cirrhosis combined with oesophageal varices was 0.23, with a corresponding sensitivity and specificity of 0.861 and 0.467, respectively.

### 3.6. Predictive Value of APRI, AAR, FIB-4, and S Index in Severe Oesophageal Varices (Shown in Figure 2)

ROC curve analysis showed that the AUC values of APRI, AAR, FIB-4, and S index in predicting severe oesophageal varices were 0.729, 0.648, 0.673, and 0.695, respectively (shown in Table 4). The optimal demarcation point of blood APRI in predicting cirrhosis combined with oesophageal varices was 1.4, with the corresponding sensitivity and specificity being 0.939 and 0.600, respectively. The optimal demarcation point of AAR in predicting cirrhosis combined with oesophageal varices was 1.3, with a corresponding sensitivity and specificity of 0.394 and 0.933, respectively. The optimal demarcation point of FIB-4 in predicting cirrhosis combined with oesophageal varices was 113.4, with a corresponding sensitivity and specificity of 0.939 and 0.533, respectively. The optimal demarcation point of the S index in predicting cirrhosis combined with oesophageal varices was 0.27, with a corresponding sensitivity and specificity of 0.861 and 0.467, respectively.

### 3.7. Nonconditional Multivariate Logistic Regression Analysis of APRI, FIB-4, and S Index for Oesophageal Varices

According to whether cirrhosis was combined with oesophageal varices, which was a two-class response variable, nonconditional logistic regression analysis was used to construct a predictive model for the three values. The introduction criterion was* P* < 0.05, and the elimination criterion was* P* >0.10. As a result, APRI and FIB-4 could be used as an independent predictive model for cirrhosis combined with oesophageal varices (shown in Table 5).

### 3.8. Nonconditional Multivariate Logistic Regression Analysis of APRI, AAR, FIB-4, and S Index for Severe Oesophageal Varices

According to whether cirrhosis was combined with severe oesophageal varices, which was a two-class response variable, nonconditional logistic regression analysis was used to construct a predictive model for the four values. The introduction criterion was* P* < 0.05, and the elimination criterion was* P* >0.10. Consistently, APRI and FIB-4 could be used as independent predictive models for cirrhosis combined with severe oesophageal varices (shown in Table 6).

## 4. Discussion

Oesophageal variceal bleeding is the most lethal complication of cirrhotic portal hypertension. However, in most cirrhosis patients, no obvious clinical manifestations are present, even at decompensation period. This is especially true of oesophagogastric varices, which is difficult to detect if gastroscopy is not performed. Gastroscopy and portal vein pressure testing harbour a relatively good predictive value for the degree of oesophagogastric varices, but they are invasive procedures that are not easily tolerated by patients due to the pain. In addition, their safety is relatively low; hence, their clinical application is limited [3, 9]. In recent years, the noninvasive prediction of oesophagogastric varices has become a hotspot of research. In consideration of the emergence of portal hypertension due to the progression of hepatic fibrosis, noninvasive biochemical markers of hepatic fibrosis have been used to predict the incidence rate of oesophageal varices in patients with cirrhosis [10]. The noninvasive serological hepatic fibrosis score has the advantages of accurate data, good repeatability, and little effect on patients, which can avoid sampling errors and interpretation errors of imaging examinations; however, its evaluation effect is not clear yet.

Cheung et al. [11] demonstrated that glutamic-oxaloacetic transaminase/glutamic-pyruvic transaminase and AAR index > 1 generally indicate the occurrence of cirrhosis. Iwata et al. [12] reported that AAR is associated with the severity of oesophageal varices. In this study, there was no significant difference in the AAR index between cirrhosis without oesophageal varices and cirrhosis combined with oesophageal varices. In the prediction of severe oesophageal varices, the AUC value of AAR was the lowest of the four scores. Hence, we demonstrate that the AAR index harboured limited predictive value for oesophageal varices.

The APRI index and FIB-4 are two classic scores and harbour good diagnostic efficiency for cirrhosis [13–15]. In this study, the AUC value of APRI for oesophageal varices and severe oesophageal varices was higher than those of the other three indexes. Notably, the AUC in the prediction of severe oesophageal varices was > 0.7 (AUC = 0.729) and the negative predictive value (NPV) was 0.963. In severe oesophageal varices, an APRI value < 1.4 was only detected in two patients, accounting for 6% of the severe oesophageal varices. Therefore, APRI > 1.4 can be used as a reference indicator for the early intervention of severe oesophageal varices. Although the FIB-4 index was found to be an independent predictor of oesophageal varices in an unconditional logistic regression analysis, the AUC value was less than 0.7; hence, FIB-4 failed to harbour a clinical diagnostic value.

The S index was proposed by Chinese scholars. In this study, the AUC value of the S index was higher than that of the FIB-4 index. However, the S index was not detected as an independent predictor of oesophageal varices by the unconditional logistic regression analysis (all* P* > 0.01). The calculation formula was 1 000×GGT/(PLT×albumin^2^). Of the albumin, GGT, and PLT established by this model, PLT and albumin have been confirmed to be associated with the severity of cirrhosis [6, 16]. GGT mainly originated from liver and is generated by the mitochondria of hepatocytes, confined to the cytoplasm and intrahepatic bile duct epithelium. However, GGT levels can be significantly elevated in the cases of fatty liver, alcoholic liver disease, and biliary system diseases [17, 18]. In this study, patients with alcoholic liver disease and autoimmune liver disease were included, which may have a certain impact on the experimental results. Additionally, ALT and AST are associated with the degree of liver damage. Intriguingly, GGT was replaced with ALT and AST in the formula of 1000×ALT/(PLT×albumin^2^) and 1000×AST/(PLT×albumin^2^), which revealed that the AUC value of predicting oesophageal varices was 0.681 and 0.663, respectively, and that the AUC value of predicting severe oesophageal varices was 0.718 and 0.701, respectively. All the AUC values were greater than 0.7. However, it was not considered as an independent risk factor for oesophageal varices by further unconditional logistic regression analysis. Therefore, whether the formula can be applied to the diagnosis and evaluation of the degree of fibrosis and hepatic lesions remains unknown.

Thus, in the four scoring systems, the AAR score harboured a poor diagnostic efficiency for oesophageal varices, while APRI scores have clinical value for the prediction of severe oesophageal varices. APRI>1.4 may be used as a reference index for early intervention in severe oesophageal varices. The S index cannot effectively predict the degree of oesophageal varices, which is possibly affected by the aetiology of cirrhosis. However, we demonstrated that the modified formula after replacing AST and ALT with GGT can be used as a potential predictive formula to assess the degree of cirrhosis progression.

## Figures and Tables

**Figure 1:**
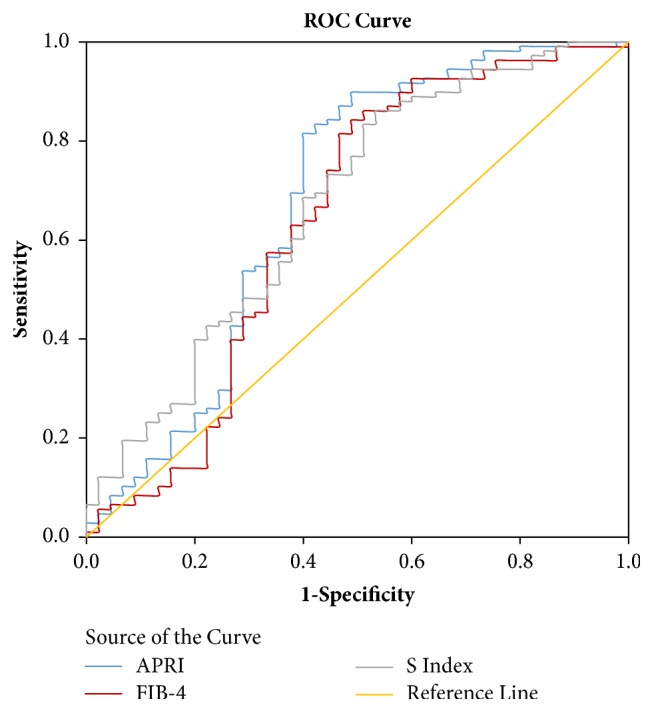
Receiver operating characteristic curve of APRI, FIB-4, and S index in diagnosis of cirrhosis combined with oesophageal varices. APRI: aspartate aminotransferase and platelet ratio; FIB-4: fibrosis index based on factor-4.

**Figure 2:**
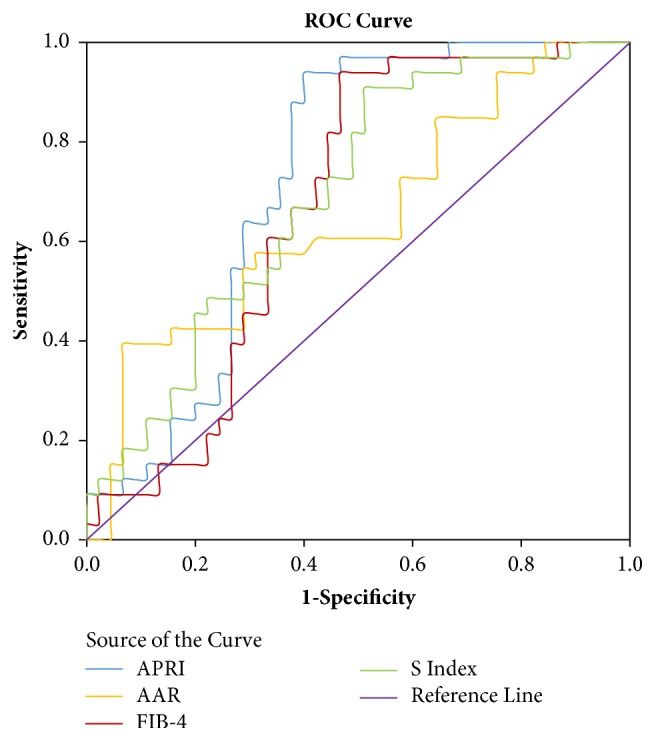
Receiver operating characteristic curve of APRI, AAR, FIB-4, and S index in diagnosis of cirrhosis combined with severe oesophageal varices. APRI: aspartate aminotransferase and platelet ratio; FIB-4: fibrosis index based on factor-4; AAR: aspartate aminotransferase-alanine aminotransferase ratio.

**Table 1:** Grading method of oesophageal varices [8].

Grade)	EV morphology (F)	EV red-color sign (RC)
mild (G1)	EV Straight or slightly tortuous (F1)	no
moderate (G2)	EV Straight or slightly tortuous (F1)	yes
	EV Swelling and distortion like snake (F2)	no
severe (G3)	EV Swelling and distortion like snake (F2)	yes
	EV Bead-like, nodular or tumor-like (F3)	yes/no

**Table 2:** General state of the 153 patients with cirrhosis.

General information	no	mild	moderate	severe
Number of cases	44	43	32	33
Mean age	51.0±12.6	48.8±12.7	52.9±11.3	47.5±9.3
Male (cases)	25	31	19	24
Female (cases)	19	12	13	9
APRI	1.0 (0.6,3.5)	1.7 (1.1,2.9)	2.2 (1.4,4.9)	2.7 (1.9,4.2)
AAR	1.1 (0.8,1.2)	1.0 (0.8,1.4)	1.1 (0.9,1.4)	1.2 (0.9,1.4)
FIB-4	102. (55.5,562.7)	196.3 (104.9,392.2)	324.3 (165.9,708.1)	244.0 (153.3,518.1)
S index	0.3 (0.1,1.0)	0.7 (0.2,1.3)	0.9 (0.4,1.9)	0.8 (0.4,1.9)

**Table 3:** The predictive value of noninvasive serological hepatic fibrosis scoring system in cirrhosis combined with oesophageal varices.

indicator	AUC	Cut-off	sensitivity	specificity	PPV	NPV
APRI	0.681	1.29	0.815	0.600	0.832	0.578
FIB-4	0.642	103.7	0.843	0.511	0.805	0.564
S index	0.673	0.23	0.861	0.467	0.793	0.613

APRI: aspartate aminotransferase and platelet ratio; FIB-4: fibrosis index based on factor-4; AUC: area under the curve; PPV: positive predictive value; NPV: negative predictive value.

**Table 4:** The predictive value of noninvasive serological hepatic fibrosis scoring system in cirrhosis combined with severe oesophageal varices.

indicator	AUC	Cut-off	sensitivity	specificity	PPV	NPV
APRI	0.729	1.4	0.939	0.600	0.640	0.963
AAR	0.648	1.3	0.394	0.933	0.667	0.661
FIB-4	0.673	113.4	0.939	0.533	0.588	0.885
S index	0.695	0.27	0.861	0.467	0.545	0.864

APRI: aspartate aminotransferase and platelet ratio; FIB-4: fibrosis index based on factor-4; AUC: area under the curve; PPV: positive predictive value; NPV: negative predictive value.

**Table 5:** Logistic regression analysis of APRI, FIB-4, and S index in prediction of cirrhosis combined with oesophageal varices.

	B	S.E.	Wald	df	Sig.	Exp(B)
APRI	.731	.264	7.647	1	.006	2.077
FIB-4	-.002	.001	8.961	1	.003	.998
S Index	.280	.205	1.855	1	.173	1.323
constant	-.187	.350	.286	1	.593	.829

**Table 6:** Logistic regression analysis of APRI, AAR, FIB-4, and S index in prediction of cirrhosis combined with oesophageal varices.

	B	S.E.	Wald	df	Sig.	Exp(B)
APRI	2.833	.762	13.814	1	.000	16.990
AAR	.016	.937	.000	1	.986	1.017
FIB-4	-.012	.003	12.302	1	.000	.988
S Index	.173	.148	1.375	1	.241	1.189
constant	-2.967	1.117	7.057	1	.008	.051

## Data Availability

The datasets used to support the findings of this study are available from the corresponding author upon request.
